# Neuroprotective and neurorestorative potential of xenon

**DOI:** 10.1038/cddis.2016.86

**Published:** 2016-04-07

**Authors:** J Lavaur, M Lemaire, J Pype, D Le Nogue, E C Hirsch, P P Michel

**Affiliations:** 1 Institut National de la Santé et de la Recherche Médicale, U 1127, CNRS, Unité Mixte de Recherche (UMR) 7225, Sorbonne Universités, UPMC Univ Paris 06, UMR S 1127, Institut du Cerveau et de la Moelle épinière, ICM, Paris, France; 2 Air Liquide Healthcare, Medical R&D Paris, Saclay Research Center, Jouy-en Josas, France

Xenon is a monatomic gas that belongs to the family of noble gases. Like other noble gases, it is characterized by a filled valence shell and therefore exhibits low chemical reactivity. Paradoxically, xenon possesses a remarkable spectrum of biological effects that are of potential clinical interest. Xenon is an approved anesthetic drug with analgesic properties.^1, 2^ In addition to that xenon is neuroprotective in preclinical models of focal and global brain ischemia, spinal cord ischemia and traumatic brain injury.^1, 2^ These neuroprotective effects are generally observed at concentrations of xenon ranging from 35 to 75%.^1, 2, 3^ Although the activation of ATP-sensitive potassium channels or of two-pore potassium channels may explain some of the neuroprotective effects of xenon, the noble gas appears to work primarily by limiting the overstimulation of *N*-methyl-d-aspartate (NMDA) glutamate receptors under excitotoxic stress conditions.^1, 4^ More specifically, xenon has been reported to compete with glycine, a co-agonist for NMDA receptor activation.^1^

Excitotoxic stress mediated through NMDA receptors is most generally associated to acute central nervous system insults such as ischemia and traumatic brain injury, but chronic, low-level overexcitation of these receptors is also a possible contributor to neuronal death in a number of chronic neurodegenerative conditions, including amyotrophic lateral sclerosis, Parkinson's disease and Alzheimer's disease (AD).^5, 6^ The implication of excitotoxic stress in AD-mediated neurodegeneration is specifically supported by studies reporting the benefits of treatments with NMDA receptor antagonists in preclinical models of the disease.^7^ Of interest, one of these antagonists memantine has also a small beneficial effect on cognitive impairment in AD patients.^7, 8^

In our work published in Cell Death Discovery,^9^ we explored for the first time the neuroprotective potential of xenon in experimental settings that mimic sustained, low-level excitotoxic stress as it may occur in the AD pathology. For that, we established cultures of neurons typically affected in this disorder, that is, cortical neurons and basal forebrain cholinergic neurons,^10, 11^ and exposed them to l-trans-pyrrolidine-2,4-dicarboxylic acid (PDC), a synthetic glutamate analog that provokes an increase in ambient glutamate through the blockade of glutamate uptake and the stimulation of its release.

When the conventional cell culture atmosphere was substituted with a gas combination, including the same amount of oxygen (20%) and carbon dioxide (5%) but 75% xenon instead of nitrogen, we observed a substantial reduction of neuronal loss induced by PDC. The noble gas argon remained inactive against PDC, pointing to the specificity of the effects of xenon in the present paradigm. Neuroprotection by xenon was mimicked by two noncompetitive antagonists of NMDA glutamate receptors memantine and ketamine, indicating that xenon might work itself by antagonizing NMDA receptors. Coherent with this view, we found that xenon remained strongly protective when NMDA, a specific agonist for NMDA receptors was used instead of PDC to trigger the death of cortical neurons. Note that we failed to demonstrate a competitive inhibition of xenon at the glycine-binding site of NMDA receptors, which is in apparent contradiction with previous reports.^1^ Yet, molecular dynamic simulation studies predict different sites of action for xenon on the NMDA receptor.^4^

Most interestingly, we found that memantine and ketamine potentiated xenon-mediated neuroprotection when each of these compounds was used at concentrations providing suboptimal rescue to cortical neurons and, most surprisingly, no rescue at all. The nature of this cooperative interaction needs to be further characterized. Yet, we may assume that it was due to the fact that xenon on one hand, and memantine and ketamine on the other hand, acted through distinct binding sites to modulate NMDA receptor activity. This type of cooperative effect is of potential clinical interest in the context of AD, as memantine is an approved drug for the treatment of this disorder.^7, 8^

Basal forebrain cholinergic neurons represent another type of neurons particularly vulnerable in the AD pathology.^11, 12^ Besides exerting true neuroprotective effects for cholinergic neurons, we established that xenon was providing trophic support for these neurons as well. This trophic effect that was most prominent in control cultures remained observable in PDC-treated cultures. The analysis of the trophic effects of xenon, revealed that the noble gas increased the size of cholinergic cell bodies and stimulated the cellular expression of the cholinergic marker protein, choline acetyltransferase transferase (ChAT). A subset of dormant cholinergic neurons was also probably the target of xenon effects as the gaseous treatment increased the number of ChAT^+^ neurons in cultures not exposed to PDC. Memantine amplified some of the effects of xenon on cholinergic neurons but was generally less efficacious than the noble gas when applied alone to the cultures. In relation with these observations, NMDA receptor blockade was reported to promote the expression of cholinergic traits during development in subsets of forebrain glutamatergic neurons.^13^ Thus, it is reasonable to believe that NMDA receptor antagonism accounted for both the trophic and restorative effects of xenon. These data are of interest as there is evidence from experimental lesions in animals and post-mortem human studies that phenotypic markers disappear early from basal forebrain cholinergic neurons vulnerable to AD pathology.^12^

In summary, present data demonstrate that the noble gas xenon has the ability to provide protection and to exert trophic or restorative effects for AD vulnerable neurons (Figure 1). Noticeably, some of the effects of xenon were improved by the AD medication memantine. Altogether, these observations are an indication that the noble gas may have potential utility for AD treatment.

## Figures and Tables

**Figure 1:**
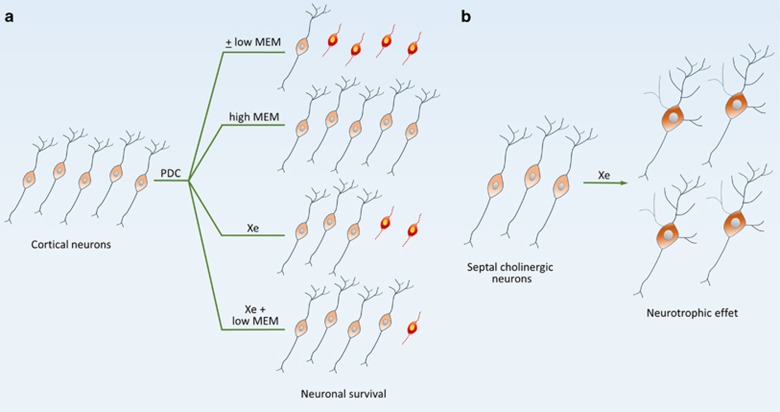
Simplified scheme describing the effects of xenon on cortical neurons and basal forebrain cholinergic neurons. (**a**) Protective effects of xenon (Xe) in cortical cultures treated with PDC to generate sustained, low-level excitotoxic stress. Xenon (75%) afforded robust but partial protection in this experimental setting. This effect was improved by a co-treatment with the noncompetitive NMDA receptor antagonist memantine (MEM), at a concentration that had no protective effect in itself (low MEM). This suggests that xenon and MEM acted cooperatively to promote neuronal survival. When MEM was used alone, at an optimal concentration (high MEM), virtually all cortical neurons were rescued. Note that a similar response profile was observed when MEM was replaced with the NMDA receptor antagonist, ketamine (not shown). (**b**) Xenon also stimulated cholinergic traits and promoted the morphological differentiation of cholinergic neurons in basal forebrain septal cultures. ±Means with or without treatment
